# Neighbourhood watch: genomic epidemiology of SARS-CoV-2 variants circulating in a German federal state, Mecklenburg-Western Pomerania, in 2020–2022

**DOI:** 10.1080/22221751.2023.2245916

**Published:** 2023-08-22

**Authors:** Christian Kohler, Jacqueline King, Lina Stacker, Katja V. Goller, Juliane Moritz, Anne Pohlmann, Neetika Nath, Ana Tzvetkova, Maximilian Rieck, Sofia Paraskevopoulou, Denis Beslic, Martin Hölzer, Stephan Fuchs, Janine Ziemann, Lars Kaderali, Martin Beer, Nils-Olaf Hübner, Karsten Becker

**Affiliations:** a Friedrich-Loeffler-Institute of Medical Microbiology, University Medicine Greifswald, Greifswald, Germany; b Institute of Diagnostic Virology, Friedrich-Loeffler-Institute—Federal Research Institute for Animal Health, Greifswald-Insel Riems, Germany; c Institute for Hygiene and Environmental Medicine and Central Unit for Infection Prevention and Control, University Medicine Greifswald, Greifswald, Germany; d Institute of Bioinformatics, University Medicine Greifswald, Greifswald, Germany; e Human Molecular Genetics Group, Department of Functional Genomics, Interfaculty Institute for Genetics and Functional Genomics, University Medicine Greifswald, Greifswald, Germany; f Genome Competence Center (MF1), Robert Koch Institute, Berlin, Germany

**Keywords:** SARS-CoV-2, whole genome sequencing, surveillance, genomic epidemiology, Mecklenburg-Western Pomerania

## Abstract

Global and even national genome surveillance approaches do not provide the resolution necessary for rapid and accurate direct response by local public health authorities. Hence, a regional network of microbiological laboratories in collaboration with the health departments of all districts of the German federal state of Mecklenburg-Western Pomerania (M-V) was formed to investigate the regional molecular epidemiology of circulating SARS-CoV-2 lineages between 11/2020 and 03/2022. More than 4750 samples from all M-V counties were sequenced using Illumina and Nanopore technologies. Overall, 3493 (73.5%) sequences fulfilled quality criteria for time-resolved and/or spatially-resolved maximum likelihood phylogenic analyses and k-mean/ median clustering (KMC). We identified 116 different Pangolin virus lineages that can be assigned to 16 Nextstrain clades. The ten most frequently detected virus lineages belonged to B.1.1.7, AY.122, AY.43, BA.1, B.1.617.2, BA.1.1, AY.9.2, AY.4, P.1 and AY.126. Time-resolved phylogenetic analyses showed the occurrence of virus clades as determined worldwide, but with a substantial delay of one to two months. Further spatio-temporal phylogenetic analyses revealed a regional outbreak of a Gamma variant limited to western M-V counties. Finally, KMC elucidated a successive introduction of the various virus lineages into M-V, possibly triggered by vacation periods with increased (inter-) national travel activities. The COVID-19 pandemic in M-V was shaped by a combination of several SARS-CoV-2 introductions, lockdown measures, restrictive quarantine of patients and the lineage specific replication rate. Complementing global and national surveillance, regional surveillance adds value by providing a higher level of surveillance resolution tailored to local health authorities.

## Introduction

At the end of 2019, a novel betacoronavirus designated severe acute respiratory syndrome coronavirus 2 (SARS-CoV-2) was identified as the causative agent of unusual viral pneumonia in the city of Wuhan, China [1]. This novel coronavirus disease, also known as coronavirus disease 2019 (COVID-19), has spread all over the world with currently (25 January 2023) 664,618,938 cumulative infections and with a death toll of around 6,722,949 million (https://covid19.who.int/). In Germany, SARS-CoV-2 was first identified near Munich, Bavaria, on 27 January 2020 [2]. To date (25 January 2023) 37,701,193 cases with a death toll of 165,001 have been confirmed in Germany (https://www.rki.de/). To effectively combat this deadly disease, genomics has contributed to understand the viral origin, outbreak dynamics and transmission and has proficiently guided the public health response to COVID-19 [3]. Additionally, whole genome sequencing enabled an observation of the SARS-CoV-2 evolution in real-time and led to the discovery of further virus variants with affected different virus’s properties. Variant under Monitoring (VUM) are variants with genomic changes that affected virus characteristics like increased growth compared to the wild type virus but the phenotypic or epidemiological impact remains unclear. Variant of Interest (VOI) are SARS-CoV-2 variants accumulating genetic changes that are predicted or known to influence the transmissibility, virulence and detectability leading to growth advantages compared over other circulating variants which lastly increasing relative prevalence alongside increasing number of cases over time. Variant of Concern (VOC) are associated with a moderate or high level of confidence to lead to an increased clinical disease severity or decrease the effectiveness of available vaccines or leading very likely to an overload of the healthcare system (https://www.who.int/publications/m/item/updated-working-definitions-and-primary-actions-for–sars-cov-2-variants). Besides global and national efforts of SARS-CoV-2 surveillance and analysis, the use of sequencing data for regional and local monitoring of outbreak transmission chains has profoundly aided disease control and prompted response time, in particular for on-site elected representatives, health care authorities, and the management of clinical and nursing facilities. In January 2021, the ongoing project “CoMV-Gen” was brought to life to produce robust and specific SARS-CoV-2 sequencing data for the federal state of Mecklenburg-Western Pomerania (M-V), Germany (www.comv-gen.de) comprising mainly rural areas in the North-Eastern part of Germany. Here, the results of this full-genome data acquisition and collection are presented and analyzed together with the associated metadata over 16 months (November 2020–March 2022). Thereby, the distribution of SARS-CoV-2 variants in M-V was closely examined, giving insights into spread and evolution of SARS-CoV-2 on a federal state level.

## Material and methods

### Structure of the CoMV-Gen project and study subjects

The CoMV-Gen project study group (www.comv-gen.de) orchestrates the collection, diagnostics, sequencing, and analyses of the SARS-CoV-2 surveillance in M-V in collaboration with the health departments of all eight districts and at least 13 laboratories which supplied the viral RNA, quantitative RT-PCR (qRT-PCR) results and/ or sequencing data. In total, 3493 samples were subjected to sequencing and phylogenetic analyzes. For details, please see supplemental material.

### Nucleic sampling, isolation and qRT-PCR

Viral RNA was extracted from nasopharyngeal swabs using different kits adjusted to detect the circulating variants during the time course. For details, see supplemental material.

### SARS-CoV-2 amplicon-based sequencing, bioinformatical analysis and used software

The RNA samples were analyzed either on Illumina or Oxford Nanopore Technologies platforms. SARS-CoV-2 consensus sequences were generated using different pipelines as described in supplemental material. The consensus sequences and accompanying metadata for the samples were uploaded to the European Nucleotide Archive (ENA) with accession number PRJEB59319. Consensus sequences were analyzed using Nextstrain [4], Pangolin [5], MAFFT v7.490 [6], RAxML v.8.2.12 [7], Augur [4], Auspice [4], TMEV 4.9.0 [8], Prism 5 (GraphPad) and Excel (Microsoft). For details, please see supplemental material.

## Results

### Sample size and structure

**Figure 1.**
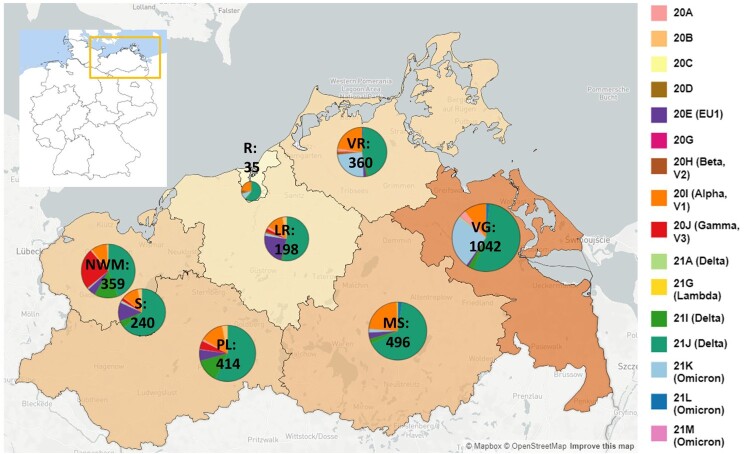
SARS-CoV-2 clades in M-V in the time period from November 2020 till March 2022. Map of M-V with the individual SARS-CoV-2 clades distribution and the total number of sequences per county. VR Vorpommern-Rügen, VG Vorpommern-Greifswald, R Rostock, LR – Landkreis Rostock, MS – Mecklenburgische Seenplatte, NWM Nordwestmecklenburg, S Schwerin, PL Parchim-Ludwigslust

In total, more than 4750 SARS-CoV-2 samples were sequenced from November 2020 until March 2022 with 3493 (73.5%) full-genome sequences fulfilling quality criteria (see Supplemental Material and Methods) for phylogenetic and spatio-temporal analyses. Respectively, 3144 (90.0%) samples belonged to residents of M-V and 349 samples were assigned to patients who did not have their main residence in M-V, but were sampled in the federal state. The majority of the sampled non-M-V residents could be traced back to the neighbouring federal state Brandenburg (n = 238; 68.2% of non-M-V residents). The remaining 85 patients were registered in other federal states of Germany (Table S1). Six samples originated from patients of other European countries and one patient originated from Egypt, Africa (Table S1). Another 19 samples were drawn from ship crews in the harbours of Rostock and Wismar, but could not be assigned to their original main residence. The infrastructure of the CoMV-Gen project (Figure S1) enabled almost even sample coverage of the entire federal state M-V (Figure 1).

### Phylogenetic analysis confirmed the successive appearance of global clades, but with a time delay

**Figure 2.**
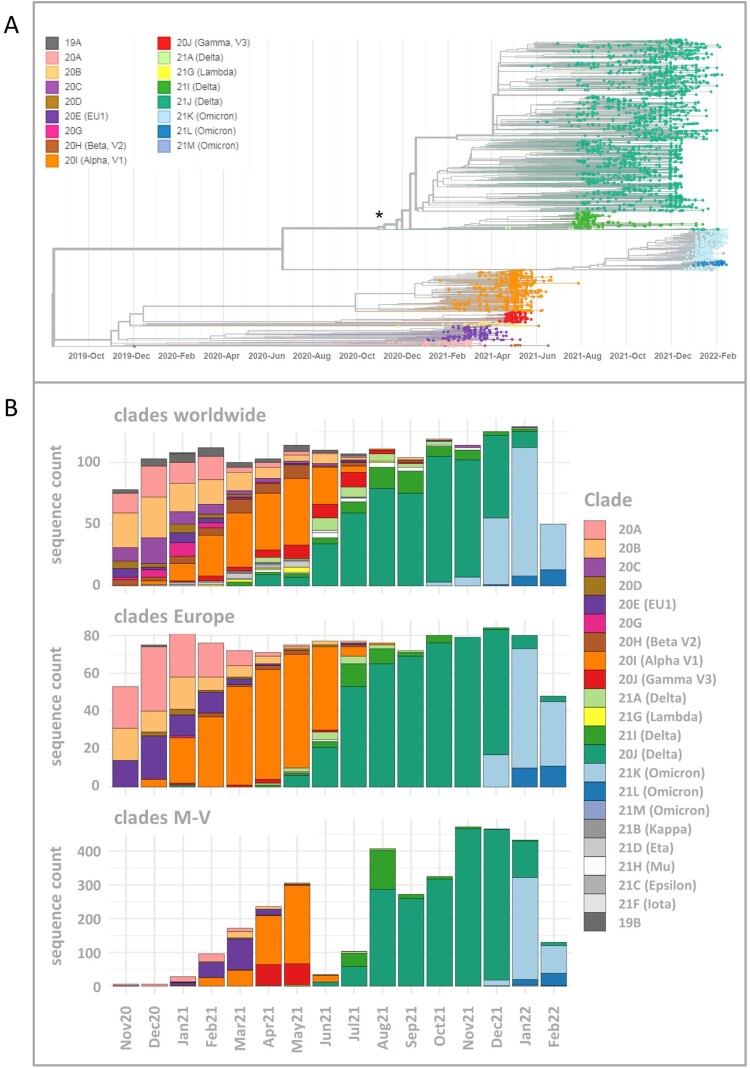
Time-resolved maximum likelihood phylogeny of 3493 in M-V sampled sequences and their comparison of SARS-CoV-2 clades circulating in Europe and worldwide during the same period. A. SARS-CoV-2 sequences were aligned by MAFFT v7.490 and the resulting multiple sequence alignment (MSA) was used to generate a corresponding phylogenetic tree using RAxML v.8.2.12. We used the WUHAN MN908947.3 as the outgroup reference sequence. To generate and visualize the corresponding time tree we utilized Nextstrain tools Augur and Auspice. The legend on the left shows the colours of the Nextstrain nomenclature clades shown in the phylogentic tree. The asterisk marks the start point of this study. B. Comparison of SARS-CoV-2 clades circulating in M-V with those found in Europe and worldwide over the same period from November 2020 to February 2022. In total, 1177 European and 1687 worldwide randomly picked sequences were downloaded from GISAID (Table S2) and compared with 3493 in M-V sampled sequences.

Including SARS-CoV-2 sequences (n = 3493) derived from residents and non-residents enabled a detailed view of all virus clades and lineages detected in M-V (Figure 2A and Table S1). We observed a similar virus clade distribution over all counties, whereby most viruses belonged to the clades of Delta (n = 2163), Alpha (n = 472), Omicron (n = 460) and EU1 (n = 164) (Table S1). The Gamma clade (n = 124) was mostly detected in the western counties of M-V (Figure 1). We identified 16 different Nextstrain clades in which 116 different Pangolin virus lineages were recovered (Table S1). The largest Pangolin virus lineage diversity was found in the Delta clade (n = 82) followed by 20A (n = 10), 20B (n = 7), 20E (n = 5), Omicron (n = 4) and the remaining clades with one to three lineages (Table S1). A time-resolved phylogenetic analysis showed successive occurrence of virus clades as determined worldwide (Figure 2A). Isolates of the second wave belonged to the Nextstrain clades 20A-H (November 2020 until March 2021), followed by Alpha and Gamma (April–June 2021). The third wave (July–December 2021) was dominated by Delta and lastly a fourth wave (January 2022 onwards) was monopolized by Omicron. This time line is almost consistent with the pandemic described worldwide, but we observed a delay in the occurrence of these clades (Figure 2B). Compared to the worldwide trend during this period, almost all variants appeared about one to two months later in M-V, in particular the variants of concern (VOCs) including the Alpha and partly the Delta and Omicron clades (Figure 2B). The time lag compared to European trends constitutes roughly one month. Interestingly, the Alpha clade in M-V disappeared promptly in June, but could be detected in Europe and worldwide until August/ September. The proportion of different clades within the Delta and Omicron groups is comparable to the global and European data (Figure 2B). Within Delta, clade 21J dominated, followed by 21I and 21A. The Omicron clade 21K dominated primarily prior to 21L and 21M.

### Spatio-temporal distribution of the virus clades in M-V

**Figure 3.**
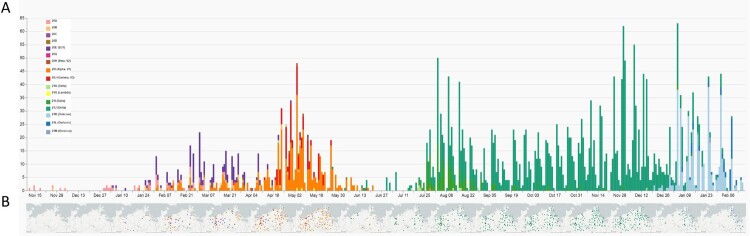
Spatio-temporal distribution of SARS-CoV-2 clades in Mecklenburg-Western Pomerania analyzed by genetic analysis of sequences generated within the framework of CoMV-Gen. A: Numbers of sequences coloured by clades level week-by-week and B geographical distribution of clades in M-V month-by-month.

Molecular surveillance of SARS-CoV-2 viruses in this project started in November and December 2020, however, the source of samples was initially limited to the eastern M-V with few samples (n = 12) (Figure 3, Table S1). Subsequent sequencing revealed uniform virus types belonging to clades 20A and 20B. By January 2021, integration of the other participating laboratories was completed, reflected in a broader distribution of samples from all regions of M-V (Figure 3). The identified clades included 20A, 20B, 20C, and 20E. By the end of January 2021, the first Alpha (20I) sequences were detected.

The pattern of viral clades in February 2021 (20A, 20B, 20E, Alpha(20I)) and March 2021 (20A, 20B, 20D, 20E, 20G, Alpha(20I)) were comparable. Until the end of March 2021, clade 20E dominated the pandemic. Interestingly, most sequences of the 20E clade were found in the western counties of M-V. Alpha was the dominant virus variant in M-V in April (20B, 20E, Beta (20H), Alpha (20I)) and May (20B, 20E, Beta(20H), Gamma (20J), Lambda (21G)). At the end of April, the first Delta clade 21A virus and at the end of May the first Alpha clade 21J case was detected (Table S1 and Figure 3).

As mentioned above, a regional outbreak of a Gamma (20J) variant was detected in western M-V in April and May 2021. This regional outbreak included cases in nursing facilities and among patients, family members, and staff (Figure S2). Originating in a nursing home, the Gamma (20J) variant spread across the western counties and circulated for approximately two months before disappearing in mid-May (Figures 3 and S2). However, two additional regional outbreaks with few samples of viruses from particular clades were characterized in May and confined to the western maritime island or urban regions in Vorpommern-Rügen (VR) (Beta (20H)) and the inner mainland in Mecklenburger Seenplatte (MS) (Lambda(21G)), respectively (Figure 3, Table S1). The number of COVID-19 cases drastically dropped in June with low-level circulation of the still dominating Alpha clade. From July onwards, Delta clades ultimately determined the course of the pandemic for the next months. In mid-December 2021, the first Omicron variant isolates were sequenced, with variants 21 K, 21L, and 21M rapidly taking over as the dominating clades from January 2022 onwards. Finally, at the beginning of March and April 2022, first recombinant SARS-CoV-2 strains were detected in Greifswald and Schwerin, respectively. These two isolates belonged to the XM clade and showed identical nucleotide sequences (100% identity from bp 55 to bp 29,713), emerging from the two circulating donor strains BA.1.1 and BA.2 (Figure S3A–C).

### Prevalence of SARS-CoV-2 lineages in M-V

**Figure 4.**
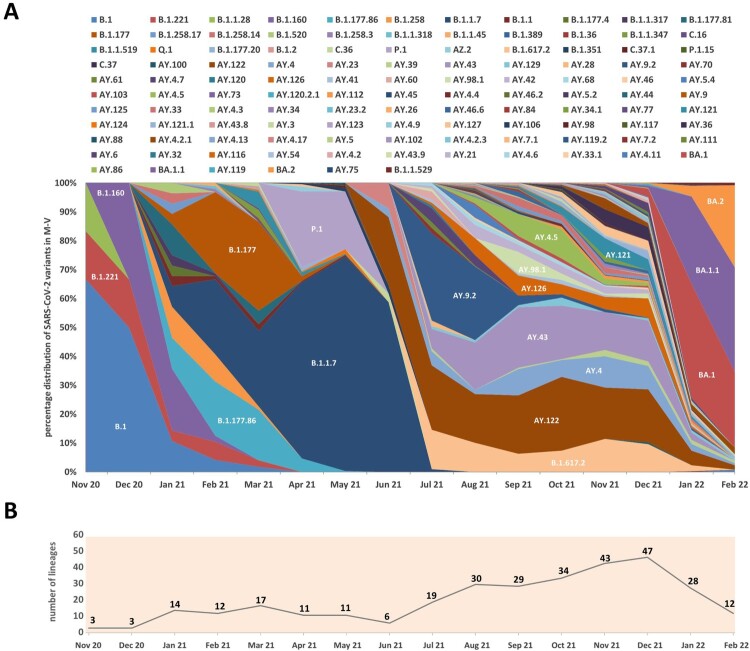
Relative distribution of SARS-CoV-2 lineages isolated in Mecklenburg-Vorpommern from November 2020 to February 2022 based on whole-genome sequencing. A. Shown is the relative lineage distribution [%] over 3493 sequences obtained in M-V. The PANGOLIN lineage system (https://cov-lineages.org/) for variants was used. The colours represent the different SARS-CoV-2 lineages. For easier orientation, SARS-CoV-2 lineages with higher proportions were marked in the diagram with white font. In addition, individual SARS-CoV-2 lineages are also listed in the legend according to their chronological course (from top left to bottom right). B. Presentation of the absolute number of SARS-CoV-2 lineages identified per month.

We classified all sequences into lineages and analyzed their distribution over the study period (Figure 4A and Table S1). The trend towards increasing identification of different SARS-CoV-2 lineages per month over the entire period was striking (Figure 4B). The first peak of 17 distinctly identified lineages was seen in March 2021 but declined to six distinct lineages by June 2021. Especially during the third pandemic wave dominated by Delta, up to 47 distinct lineages per month (December 2021) were identified in M-V. With the first appearance of the Omicron variants in December 2021, the large diversity of lineages disappeared and dropped immediately to 28 in January 2022 and 12 in February 2022 (Figure 4B). The ten most sequenced lineages belonged to B.1.1.7, AY.122, AY.43, BA.1, B.1.617.2, BA.1.1, AY.9.2, AY.4, P.1 and AY.126 (Table S1). Together they accounted for more than two-thirds of all sequences (n = 2375, 68.0%).

**Figure 5.**
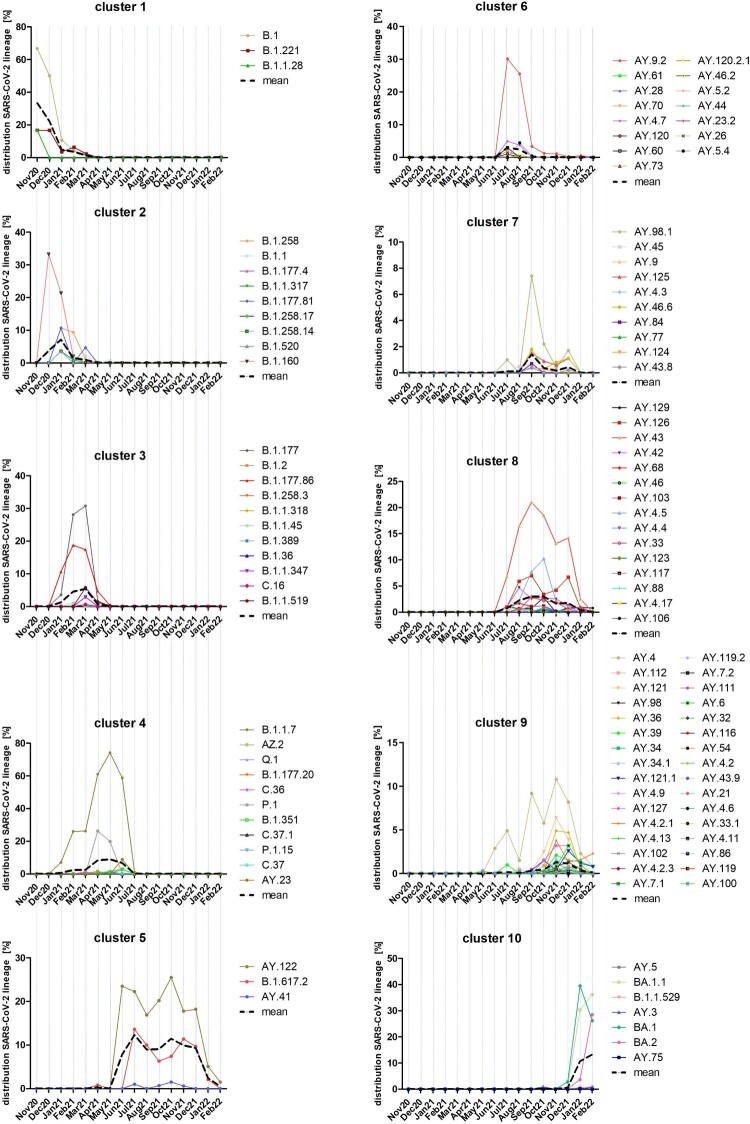
Time-resolved k-mean/median-clustering (KMC) of SARS-CoV-2 lineages identified from November 2020 to March 2022 in M-V. 3493 sequences were assigned to 116 different PANGOLIN lineages and combined with the date of infection. Afterwards a KMC was used to find lineages with a similar time course of occurrence. Shown are the distributions of every lineage in [%] over all sequences. The KMC was performed by the MultipleExperiment Viewer 4.9.0 (TMEV 4.9.0) with following parameters: KMC mode: calculated means, cluster: 10, Iterations: 50, HCL: no linkage, Pearson correlation.

A k-mean/median clustering (KMC) analysis of all lineages’ time courses elucidated a common appearance or disappearance (Figure 5). By using 10 clusters for this analysis, clusters 1–4 appeared from November 2020 to July 2021. In November and December 2020, the lineages of cluster 1 (B.1, B.1.221 and B.1.1.28) dominated, but B.1.160 rapidly increased and replaced the lineages of cluster 1 already in December 2021 as did further B.1 lineages of cluster 2 (B.1.258.X and B.1.177.X variants, B.1.520 and B.1.1.317) in January 2021. Cluster 2 lineages determined the outbreak from January 2021 to February 2021. Some lineages of cluster 3 (B.1.177, B.1.177.86) were already identified in January 2021 but reached their maximal mean from February 2021 to March 2021 before decreasing in April 2021 close to zero. From April to June 2021, cluster 4 lineages, mainly the two VOCs Alpha (B.1.1.7) and Gamma (P.1), determined the pandemic. Additional nine lineages of cluster 4 were identified, but only with low proportions. The third wave started in July 2021 with a dominance of Delta lineages which could be divided into five clusters (5–9). Lineages AY.122 and B.1.617.2 could be found constantly in high numbers over the complete time period from July to December 2021. Together with AY.41, which was identified with low numbers at the same time, they formed cluster 5. The lineages of clusters 6 and 7 appeared mainly from July to August 2021 (cluster 6) or September to November 2021 (cluster 7). Another 15 lineages belonged to cluster 6 with the dominating lineage AY.9.2. Cluster 7 was dominated by AY.98.1 and included another nine lineages. Common to both clusters was their short peak of only one to two months. Cluster 8 lineages appeared similar to cluster 5 lineages, but the mean was clearly lower and the peak was from September to October 2021. The dominating lineage was AY.43 followed by AY.126, AY.4.5 and AY.42. Lineages of cluster 9 appeared mainly from November to December 2021. This cluster contained 32 different lineages and was dominated by AY.4, AY.121 and AY.36. Cluster 5, 6, 8 and 9 lineages were rapidly displaced by cluster 10 lineages in January and February 2022, which mainly belonged to the Omicron lineages. The dominating lineages were BA.1, BA.1.1 and from February 2022 onwards, BA.2. Lineages AY.5, AY.3 and AY.75, which also belonged to cluster 10, showed a similar time course. However, their proportion was lower than 1% (Table S1). In general, some Delta lineages of clusters 5, 6, 8 and 9 were identified in low but strongly decreasing numbers in January and partly in February 2022 (Figures 4A and 5). Their proportion was about 25% in January and decreased further to 8% in February.

### SARS-CoV-2 samples of non-M-V residents

**Figure 6.**
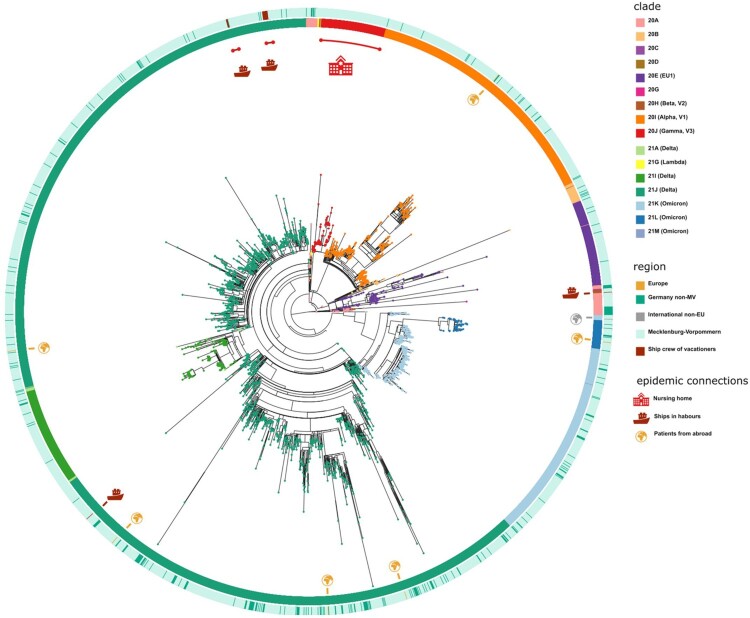
Spatially-resolved maximum likelihood phylogeny of M-V sampled sequences. SARS-CoV-2 sequences were aligned by MAFFT v7.490 and the resulting MSA was used to generate a corresponding phylogenetic tree using RAxML v.8.2.12. We used the WUHAN MN908947.3 (19A) as the outgroup reference sequence. For the visualization we used the ggtree package. Tip nodes are coloured by clades. The inner circle shows the Nextstrain nomenclature clades and the outer circle describes the corresponding region of origin. Decrypted epidemic connections are marked with pictograms.

**Figure 7.**
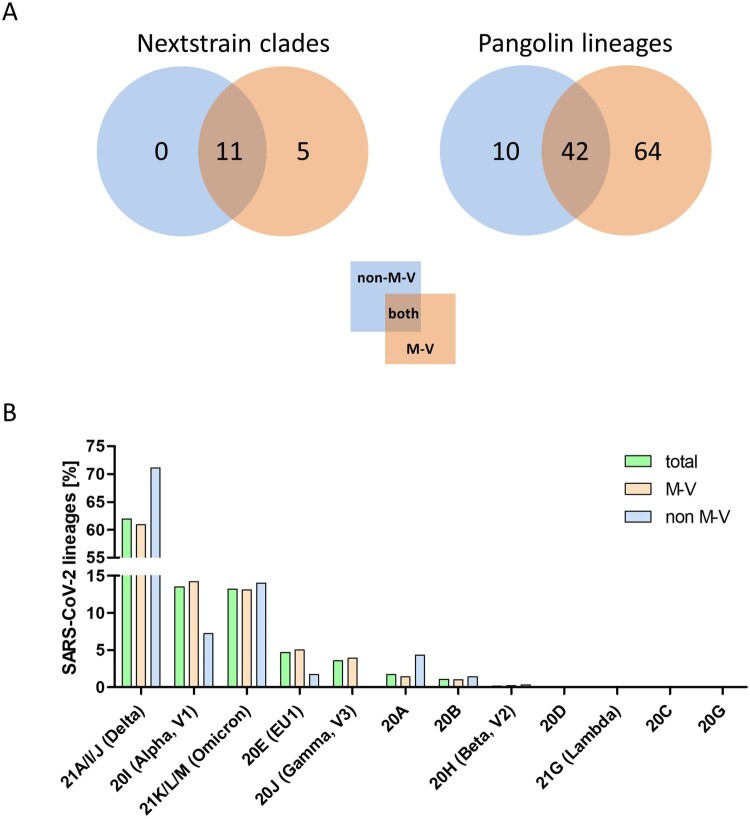
Distribution of SARS-CoV-2 clades and lineages of non-M-V and M-V residents and the proportion of the SARS-CoV-2 lineages over all clades and/or WHO label. A. Distribution of SARS-CoV-2 clades and lineages of non-M-V and M-V residents. The clades were determined by Nextstrain (https://nextstrain.org/sars-cov-2) and the lineages were taken from Pangolin (https://cov-lineages.org/resources/pangolin.html). The distributions are shown in VENN diagrams including all single clades (left side) or all single lineages (right side). B. Shown is the relative distribution of SARS-CoV-2 lineages per clade over all samples drawn in M-V (total, n = 3493), main residents of M-V (M-V, n = 3144) and non-M-V residents (non-M-V, n = 349). X-axis shows the Nextstrain clades and /or WHO label (Alpha, Beta, Gamma, Delta, Lambda, Omicron). For clarity, Nextstrain clades belonging to one WHO label (Delta and Omicron) have been grouped together.

**Figure 8.**
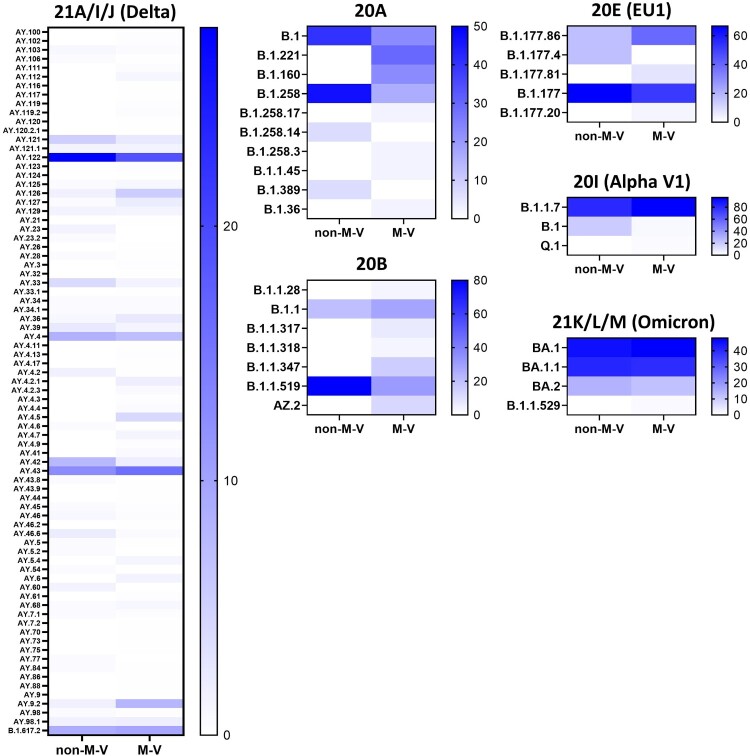
Heatmap showing the proportion of the SARS-CoV-2 lineages within a clade and/or WHO label (Alpha, Delta, Omicron). Percentages were calculated from the number of sequences per lineage and clade/WHO label relative to the total number of all samples of each clade and/or WHO label within the respective group. Not shown are the data of the clades 20C, 20D, 20G, 20H (Beta), 20J (Gamma) and 21G (Lambda) because of their low numbers of samples per lineages and clade or the sequences were only found in one group (e.g. 20J (Gamma)). The strength of the blue colour corresponds to the percentage of the SARS-CoV-2 line per clade and/or WHO label and can be evaluated in the respective legend. non-M-V: non-M-V residents, M-V: M-V residents

The 349 samples originated from non-M-V residents includes samples from patients of directly adjacent federal states, but also more distant federal states, from abroad and ship crews. Spatially-resolved maximum likelihood phylogenetic analysis showed a particularly large number of sequences of non-M-V residents clustered in the Delta clade (Figure 6). Likewise, clade 20A demonstrated a relatively high number of samples of non-M-V residents. Few sequences have been found in the Alpha and 20E (EU1) clades. A similar uniform distribution was identified for Omicron variants and 20B and 20H clades. Further, we compared the distribution of all Nextstrain-based SARS-CoV-2 clades and Pangolin-based lineages between non-M-V and M-V residents (Figure 7A). Overall, eleven out of 16 clades could be found in both groups, five clades (20H, 21G, 20C, 20G and 20J) were only found among the M-V residents, but no additional clades have been identified in the non-M-V resident group (Figure 7A, Table S1). In comparison, ten SARS-CoV-2 lineages were identified only in samples of non-M-V residents consisting of only 14 sequences and usually detected as singular cases (Table S1 and Figure 7A). While 42 lineages were found in both groups, 64 lineages were only detected in the M-V residents group (Table S1 and Figure 7A). The relative virus lineage distribution was similar but not equal between samples from M-V and non-M-V residents (Figure 7B). We observed slightly higher proportions of the Delta (21A/I/J), and the 20A variants and fewer proportions of the Alpha (20I), and 20E (EU) clades in the non-M-V resident group. In comparison to the high number of Gamma (20J) variants detected in M-V residents, surprisingly no Gamma lineages were detected in non-M-V residents. A more detailed analysis of each clade and/or the WHO label revealed differences in their relative composition (Figure 8). For example, in clade 20A the lineages B.1 and B.1.258 were dominant among non-M-V residents. In contrast, we found higher variability in the M-V group. Similar results could be found for the 20B and the Delta clade. Lineage B.1.1.519 of the 20B clade and AY.4, AY.33, AY.42, AY.121 and AY.122 of the Delta clade showed higher proportions in non-M-V residents, whereas the proportions of AY.9.2, AY.43, AY.4.5 and AY.126 were increased in M-V resident samples (Figure 8). Similar relative compositions could be found for 20E, Alpha and Omicron clades. However, of 349 non-M-V residents, 26 patients came from abroad. About 85% (n = 22) of their virus sequences were assigned to the Delta clades, with lineages B.1.617.2, AY.122 and AY.23 dominating (Table S1). AY.23 was exclusively found in patients of a cruise ship, but never identified in the M-V resident group.

## Discussion

National whole genome sequencing efforts have been highly imbalanced, leading to global disparities in SARS-CoV-2 genomic surveillance [9,10]. Likewise, discrepancies on a federal state level could lead to inconsistent sequencing results. For example, approximately 350 SARS-CoV-2 complete genome sequences were submitted from laboratories in Berlin until the end of December 2020 to GISAID, but in same time period only seven SARS-CoV-2 sequences were transferred from laboratories in M-V [11]. The CoMV-Gen project was initiated to permit reliable genomic SARS-CoV-2 surveillance of M-V and to overcome those diagnostic inconsistencies.

The project started during the second wave of the pandemic in Germany in November/December 2020. The identified lineages with high proportions of B.1, B.1.160 and, especially, B.1.177 plus sublineages B.1.177 and B.1.177.X typically dominated the pandemic as revealed in the other federal states of Germany, but generally also in Europe [12,13]. The dominance of the B.1 variant in November-December 2020 was due to the small sample size, which could possibly introduce some bias. Interestingly, B.1 was only found until October 2020 in the most eastern German federal states [12]. B.1, B.1.160 and B.1.177/B.1.177.X shared the substitution D614G in the Spike (S) protein, resulting in a higher viral load and improved SARS-CoV-2 fitness compared to the Wuhan isolate [14-17]. Although B.1.160 and B.1.177/B.1.177.X carried additional amino acid substitutions such as S:S477N (B.1.160) or S:A222 V (B.1.177/B.1.177.X), they did not lead to an increase in transmissibility and, thereby, to their dominance in the first quarter of 2021. However, these shifts were most likely due primarily to epidemiological factors [13]. We assume that the increase of especially B.1.177/B.1.177.X cases resulted from external introductions during the Christmas season in 2020. By contrast, in November-December 2020, the VOC Alpha spread rapidly across England and then worldwide, a lineage with many non-synonymous substitutions of immunological importance [17,18]. First Alpha variant sequences were found sporadically in M-V in January 2021 (Figures 2, 3 and 5), certainly attributed to Christmas travel, but its elevated transmissibility led to a rapid increase and finally displace of all former SARS-CoV-2 clades in April/May 2021. In April and May 2021, we additionally observed the rapid dissemination of a second VOC Gamma in the western counties. Gamma originated from Brazil, spread mainly over South America and displayed lineage-defining mutations in the S-protein, similar to the impact of B.1.1.7 on transmission, immune escape and virulence [19-21]. Originating from a nursing home, we could track the spread across the western counties of M-V in real time. Immediately initiated quarantine measures helped to end the respective outbreak. Unfortunately, it could not be determined from whom or how the Gamma variant came into M-V, but South American returnees or contacts with such are very likely. The second pandemic wave in M-V abruptly ended in June 2021 and positive cases dropped to almost zero (Figure 2), likely as a result of seasonality and increased outdoor activities [22].

In December 2020, a third VOC Delta evolved in India and later spread worldwide [23]. The Delta variants outcompeted their predecessors by decisive mutations like L452R, P681R, and T478 K in the S-protein leading to enhanced affinity of the S-protein to the angiotensin-converting enzyme-2 (ACE2), which increased its transmissibility, while decreasing vaccine efficacy compared to other VOCs such as Alpha [24]. The first Delta case in Europe was detected in the United Kingdom (UK) on 22 February 2021, which is historically strongly associated with travel to India and onward community transmission [25,26]. In M-V, the first case of Delta was detected at the end of April 2021 and the VOC displaced the Alpha variant ultimately and became dominant in June/July 2021 (Figures 3 and 5). In the following third wave, some Delta lineages (AY.122, B.1.617.2, AY.43 and AY.4) seemed to be more resilient than other Delta variants as they were detected in constant high proportions until December 2021 (Figure 5). The same observations were also made in countries surrounding Germany, further suggesting a constant introduction of these lineages to M-V during the holiday season [27-29]. Other lineages occurred in higher proportions only for one to two months (AY.9.2 July/August 2021 and AY.98.1 September 2021) and many remaining Delta strains with low proportions occurred either mainly in July (cluster 6), September (cluster 7), August-December (cluster 8) or October-December 2021 (cluster 9) (Figure 5). These data suggest that their introductions occurred successively: immediately after the second lockdown in June/July (cluster 5/ 6), after the school summer holidays in July/ August (cluster 7/ 8) and after the autumn holidays in September/October (cluster 9). The strict domestic quarantine conditions probably meant that less assertive variants were replaced quickly by newer variants, introduced during these periods due to increased (inter-) national travel activities. For the same reason, Omicron VOCs, first detected in Africa, came to M-V. Their high transmissibility caused by an unusual variety of mutations in the S-protein led to a fast and worldwide replacement of the Delta clade within two months and initiated the still ongoing fourth pandemic wave (Figures 2 and 3) [30,31]. In Europe, but also in M-V, Omicron lineages became dominant within a month (Figures 2B and 5). In February 2022, a strong increase of BA.2 was observed in M-V, which can be explained by the higher effective reproduction number than BA.1 [32]. Shortly after the end of the study period (February 2022), BA.2 conquered the majority before the now dominant BA.5 Omicron variants replaced BA.2 lineages in June 2022 [33]. To date (June 2023), groups of different recombinants, especially the XBB variants, determine the infection process worldwide (https://www.who.int/publications/m/item/weekly-epidemiological-update-on-covid-19 – 1 June 1 2023). It is therefore important to mention that the CoMV-Gen network made it possible to detect a SARS-CoV-2 recombinant (XM) in M-V very early in March 2022.

Our study period encompassed two completed waves (Alpha and Delta) and the beginning of a third wave (Omicron) of SARS-CoV-2 VOCs. We observed that the three VOCs appeared with a delay of several weeks in M-V. Especially, the Alpha VOC dominated Europe two months before taking over in M-V (Figure 2). This was certainly a result of the restricted travel regulations during the lockdown in spring 2021. Thereafter the measures were relaxed, resulting in faster entries of Delta and later Omicron lineages compared to Alpha variants, in addition to their higher transmissibility. We assume that most virus entries came from M-V residents returning from travel and/ or commuters rather than from foreigners who travelled to M-V during this period. Nevertheless, it cannot be ruled out because M-V includes popular tourist regions, albeit for national rather than international travellers. Comparison of non-M-V residents versus M-V residents elucidated only moderate SARS-CoV-2 diversity between both groups (Figures 6–8). In addition, the probability that viral lineages originated from international shipping was minimal. We observed only one lineage (AY.23) in a ship's crew, which has never been detected before in our setting.

However, our study has two limitations. First, only a restricted number of samples were analyzed which certainly led to a bias, especially at the beginning of our study. Second, we could not include all sequenced samples as the quality of 26.5% (n = 1257) of the sequences was too low for further analyzes.

In sum, this study has impressively demonstrated that the use of modern sequencing technologies in combination with a close collaboration between laboratories and health authorities is of great importance for the genomic epidemiology in particular on a regional level. This enabled us to quickly locate and track outbreaks of specific VOCs at the district level. At the same time, the close SARS-CoV-2 surveillance for the entire federal state of Mecklenburg-Vorpommern enabled us to classify it nationally and internationally. Finally, our study indicates that the combination of lockdown measures, restrictive quarantine of patients, the individual travel activities of residents and commuters and the lineage specific replication rate ultimately shaped the pandemic in M-V.

## Supplementary Material

Supplemental Material

Supplemental Material

Supplemental Material

Supplemental Material

Supplemental Material

Supplemental Material

## Data Availability

All sequence data generated for this study were deposited in the European Nucleotide Archive (ENA) with accession number PRJEB59319.
