# Supplementary material for: Neighbourhood watch: genomic epidemiology of SARS-CoV-2 variants circulating in a German federal state, Mecklenburg-Western Pomerania, in 2020–2022
Source: Emerg Microbes Infect. 2023 Aug 22;12(2):2245916. doi: 10.1080/22221751.2023.2245916 (PMC10446807; doi:10.1080/22221751.2023.2245916)
Supplement: Supplemental Material [file TEMI_A_2245916_SM3289.pdf]

Figure S3A

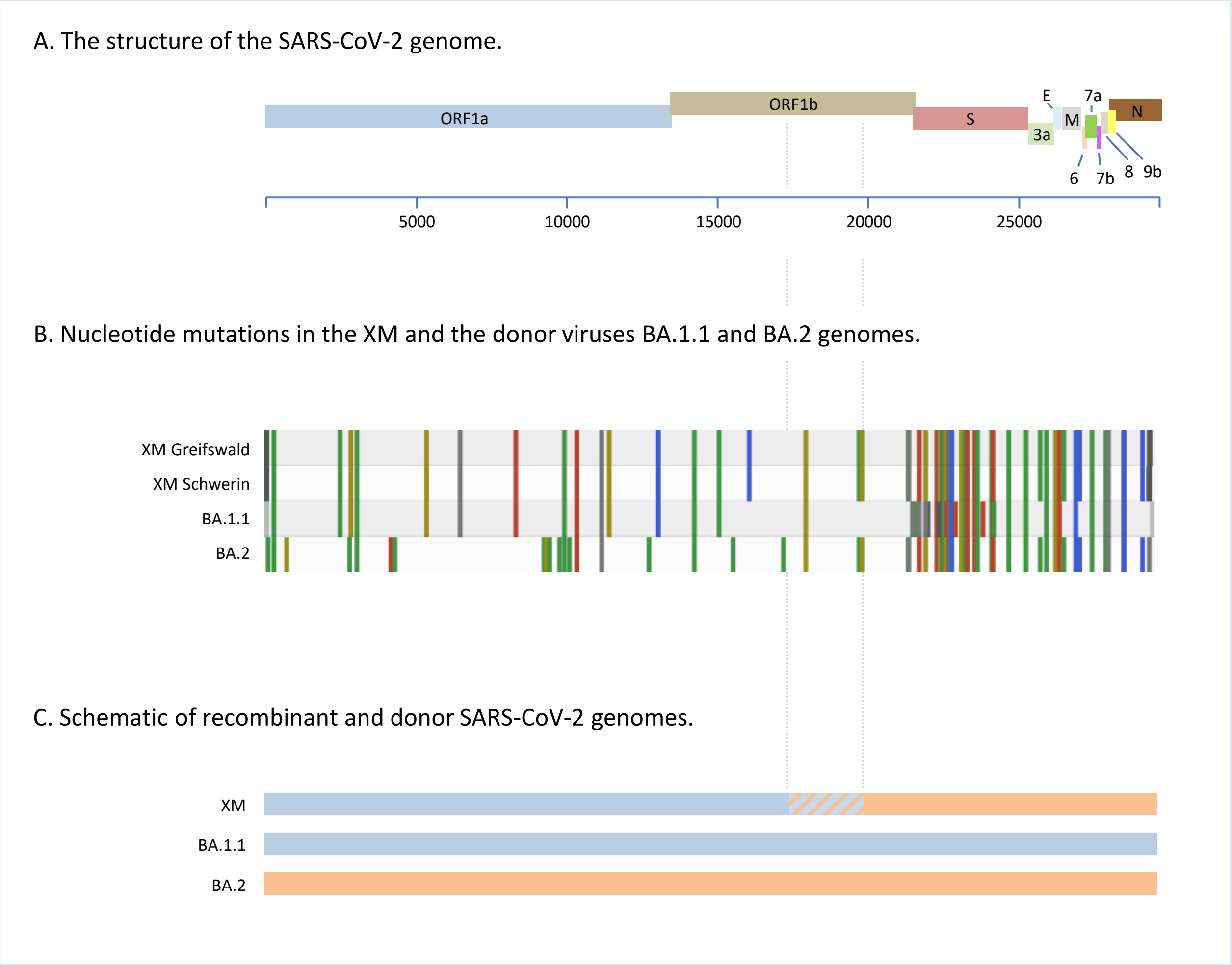

Figure S3A. Structure of the recombinant SARS-CoV-2 XM variant from Greifswald and Schwerin (M-V, Germany) and of the BA.1.1 and BA.2 donor strains. **A.** Shown is the schematic SARS-CoV-2 genome with a scale of the base pair positions (0 – 29,903). **B.** Overview over all nucleotide mutations in both XM variants, the BA.1.1 and the BA.2 variant. The consensus sequences of all strains were analyzed and visualized by Nextstrain © software (https://clades.nextstrain.org). **C.** Structure of the XM variant compared to the donor strains BA.1.1. and BA.2. The blue/orange hatched region represents the possible locus of recombination between BA.1.1 and BA.2. The likely breaking point is between NSP13 and NSP15 of ORF 1ab as previously shown by other groups (https://github.com/cov-lineages/pango-designation/issues/472). blue: BA.1.1., orange: BA.2

Figure S3B

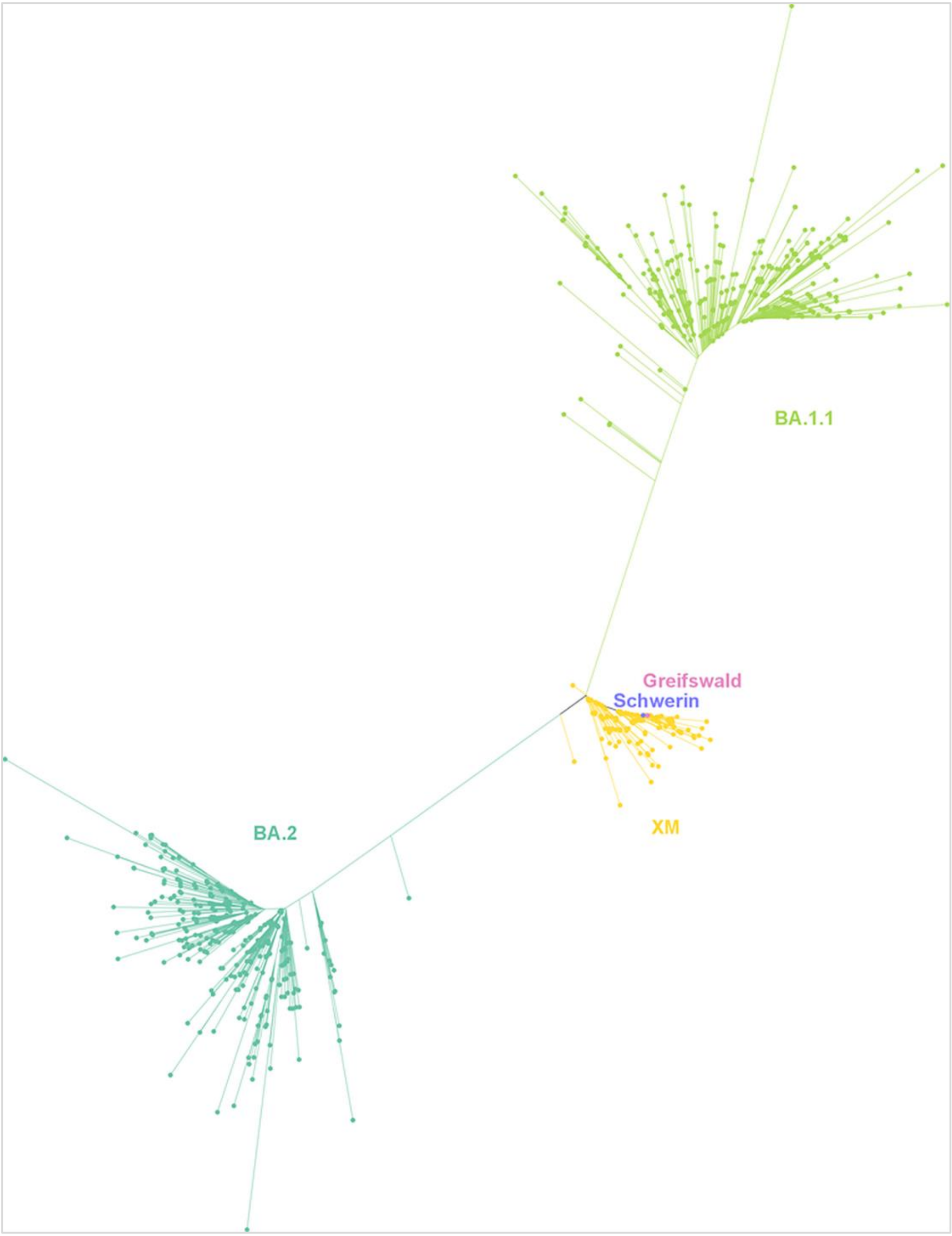

Figure S3B. Phylogenetic tree of the XM variants and the donor sublineages BA.1.1 and BA.2 for sequences obtained in Germany (available at doi.org/10.5281/zenodo.5139363). In total, 189 XM genomes (obtained on 28th April 2022) and 300 randomly selected genomes of each BA.1.1 and BA.2 sublineages were analyzed. Genomes with >3% N content and genomes causing long branch attraction effects were excluded, resulting in 734 genomes for downstream analyses (including 151 XM). Multiple sequence alignment was performed with MAFFT v.7.490 [1] in default mode. Phylogenetic tree inference was carried out in IQ-TREE multicore version 2.2.0-beta [2] with 1000 ultrafast bootstrap replicates. Visualization was done in Iroki [3]. XM variants of Greifswald and Schwerin are highlighted.

Figure S3C

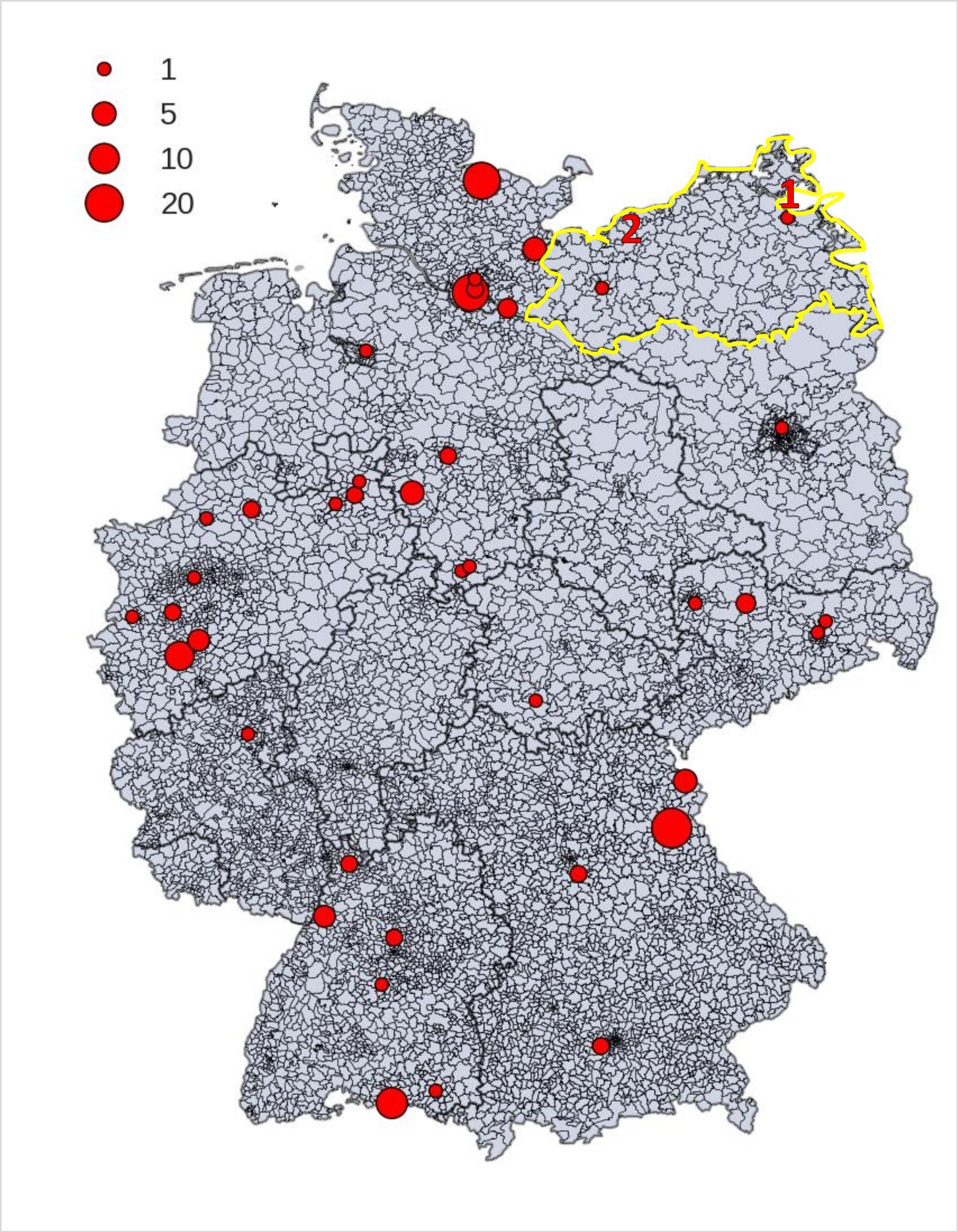

Figure S3C. Locations of primary detection of SARS-CoV-2 XM variants until 28th April 2022 in Germany. The size of the bubbles reflects the number of XM isolates per location. In total, 151 XM isolates were included after quality control (>3% N content, removal of sequences causing long branch attraction (LBA)). The border of Mecklenburg-Western Pommerania is highlighted in yellow. The illustration was created using GeoPandas v.0.10.2 (DOI: 10.5281/zenodo.3946761) and Matplotlib v.3.5.2 [4]. 1 – Greifswald, 2 – Schwerin. Metadata information for these sequences was obtained from doi.org/10.5281/zenodo.5139363. The sampling locations shown correspond to the zip codes of the primary diagnostic labs. In addition, the XM sequences were obtained via three different sampling strategies and summarized for this visualization: random (n = 87), suspect (n = 10), and unknown (n = 54).

References Figure S3A/B/C

1. Katoh K, Standley DM. MAFFT multiple sequence alignment software version 7: improvements in performance and usability. Mol Biol Evol. 2013;30(4):772-80. doi: 10.1093/molbev/mst010. PubMed PMID: 23329690; PubMed Central PMCID: PMC3603318.
2. Minh BQ, Schmidt HA, Chernomor O, Schrempf D, Woodhams MD, von Haeseler A, et al. IQ-TREE 2: New Models and Efficient Methods for Phylogenetic Inference in the Genomic Era. Mol Biol Evol. 2020;37(5):1530-4. doi: 10.1093/molbev/msaa015. PubMed PMID: 32011700; PubMed Central PMCID: PMC7182206.
3. Moore RM, Harrison AO, McAllister SM, Polson SW, Wommack KE. Iroki: automatic customization and visualization of phylogenetic trees. PeerJ. 2020;8:e8584. doi: 10.7717/peerj.8584. PubMed PMID: 32149022; PubMed Central PMCID: PMC7049256.
4. Hunter JD. Matplotlib: A 2D Graphics Environment. Computing in Science & Engineering. 2007;9(3):90-5. doi: 10.1109/MCSE.2007.55.
