# Supplementary material for: Neighbourhood watch: genomic epidemiology of SARS-CoV-2 variants circulating in a German federal state, Mecklenburg-Western Pomerania, in 2020–2022
Source: Emerg Microbes Infect. 2023 Aug 22;12(2):2245916. doi: 10.1080/22221751.2023.2245916 (PMC10446807; doi:10.1080/22221751.2023.2245916)
Supplement: Supplemental Material [file TEMI_A_2245916_SM3288.pdf]

Figure S2

A

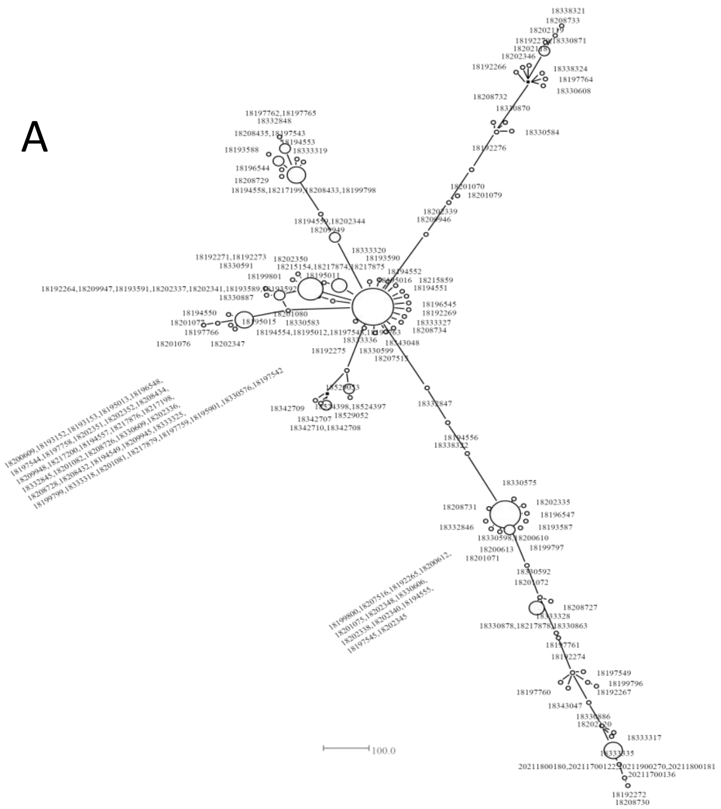

B

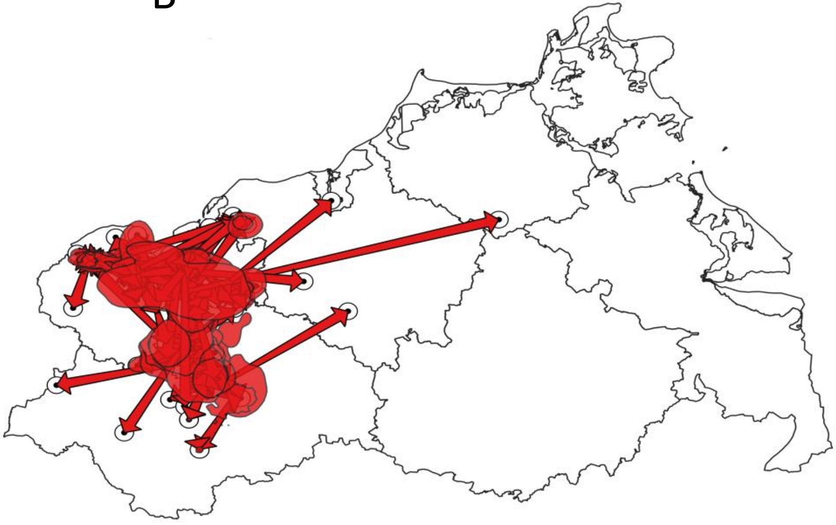

Figure S2. **A.** Median-joining networks of SARS-CoV-2 clade 20J (gamma) genomes. **B.** Connected cluster areas (polygons) with their directed spread (arrows) of SARS-CoV-2 clade Gamma genomes inferred by spatial-time phylogeography.
