# Supplementary material for: Neighbourhood watch: genomic epidemiology of SARS-CoV-2 variants circulating in a German federal state, Mecklenburg-Western Pomerania, in 2020–2022
Source: Emerg Microbes Infect. 2023 Aug 22;12(2):2245916. doi: 10.1080/22221751.2023.2245916 (PMC10446807; doi:10.1080/22221751.2023.2245916)
Supplement: Supplemental Material [file TEMI_A_2245916_SM3287.pdf]

Figure S1

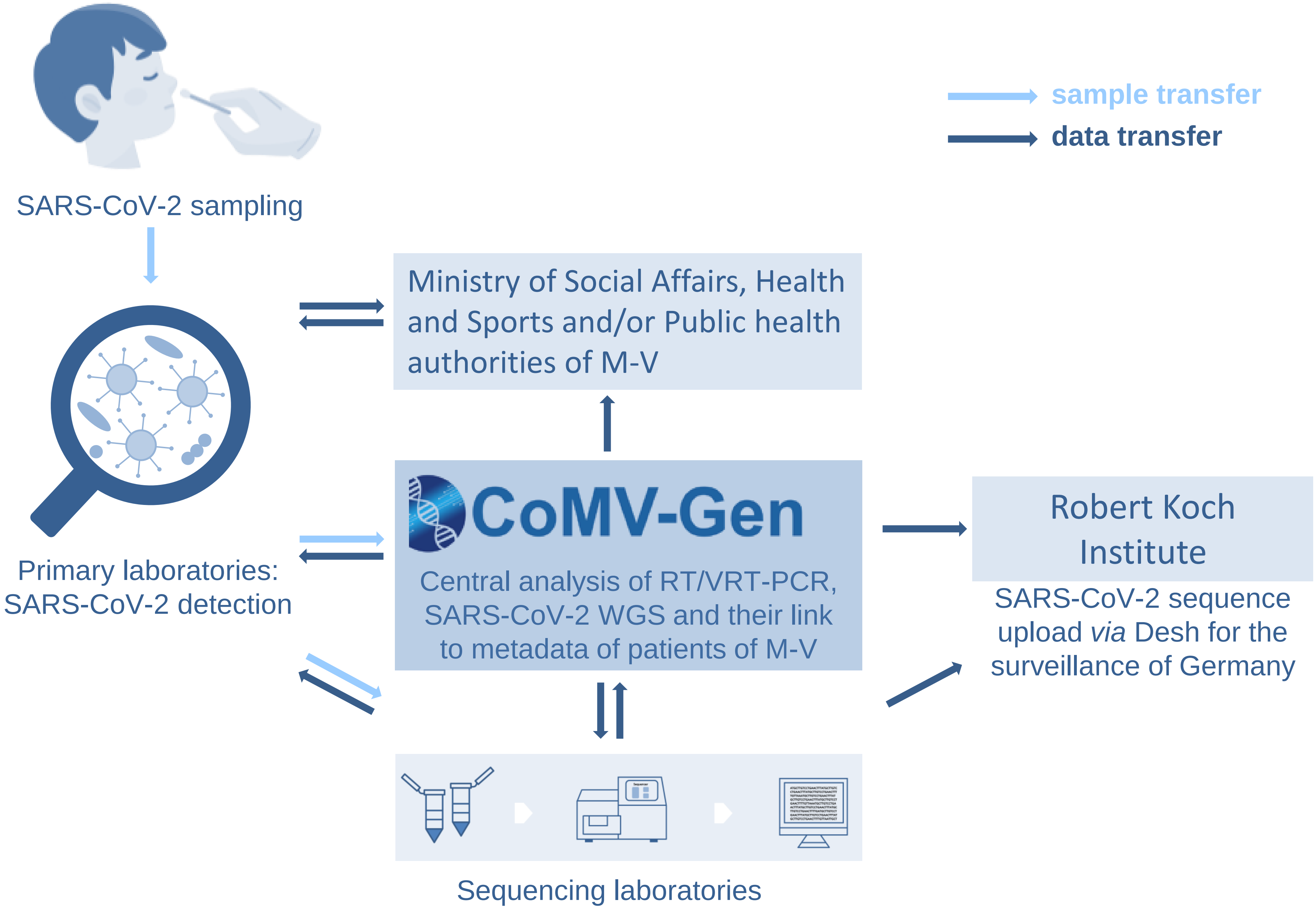

Figure S1. Schematic structure of the CoMV-Gen project. The CoMV-Gen project was founded in January 2021 at the University Medicine Greifswald connecting 13 collaborating laboratories and all local public health authorities of M-V. RNA samples were drawn throughout M-V and SARS-CoV-2 diagnostic was done by primary laboratories using RT/VRT-PCR. About 10% of all positive samples were sent to sequencing laboratories and the SARS-CoV-2 lineage was determined. All PCR data and sequencing results as well as metadata were transferred to the CoMV-Gen study group center, analysed bioinformatically and published as weekly reports in the public domain and made available to the health service and government of M-V. In addition, quality-checked SARS-CoV-2 sequences were uploaded to the RKI via Desh and the health authorities of M-V were informed about the identified virus lineages.
