# Supplementary material for: Neighbourhood watch: genomic epidemiology of SARS-CoV-2 variants circulating in a German federal state, Mecklenburg-Western Pomerania, in 2020–2022
Source: Emerg Microbes Infect. 2023 Aug 22;12(2):2245916. doi: 10.1080/22221751.2023.2245916 (PMC10446807; doi:10.1080/22221751.2023.2245916)
Supplement: Supplemental Material [file TEMI_A_2245916_SM3343.docx]

**Supplemental material**

**Material and Method**

**Sample selection and quality criteria for SARS-CoV-2 analysis**

To monitor the circulation of SARS-CoV-2 lineages in M-V, the CoMV-Gen project was founded in January 2021 in conjunction with 13 collaborating laboratories and all public health authorities of M-V. These institutions provided SARS-CoV-2 RNA samples, metadata and/or sequencing data (Figure S1). Beside their central role as the SARS-CoV-2 main surveillance unit summarizing all RT-PCR- and sequencing-based SARS-CoV-2 results in weekly reports for M-V, the CoMV-Gen project was mainly responsible for full genome SARS-CoV-2 sequencing, bioinformatic and phylogenetic analyses.

For SARS-CoV-2 confirmation, RT-PCR, Variant-PCR and/or sequencing analyzes were performed in at least 13 laboratories (table A). Samples for whole-genome-sequencing (WGS) and subsequent analyses were selected according to the following criteria: We only included samples with complete metadata such as sample collection date or SARS-CoV-2 diagnosis and patient’s city of residence represented by the zip code. Second, we included only RNA´s with RT-PCR based cycle threshold (Ct) values of < 35. After WGS, all genomes with an N content of > 10 % were excluded from any further bioinformatical and phylogenetic analysis.

Table A of all collaborating laboratories within the CoMV-Gen group.

| **Collaborating laboratory** | **Website** |
| --- | --- |
| Dietrich-Bonhoeffer-Klinikum Neubrandenburg GmbH | https://dbknb.de/ |
| Friedrich Loeffler Institute for Medical Microbiology, University Medicine Greifswald | https://www2.medizin.uni-greifswald.de/mikrobio/institut/ |
| Friedrich-Loeffler-Institute Federal Research Institute for Animal Health | https://www.fli.de/en/about-us/historie/fli-riems/ |
| Helios Kliniken Schwerin GmbH | https://www.helios-gesundheit.de/kliniken/schwerin/ |
| IMD Labor Greifswald GmbH | https://www.imd-greifswald.de/de |
| Institute for Medical Microbiology, Virology and Hygiene, University Medicine Rostock | https://imikro.med.uni-rostock.de/ |
| Labormedicus GmbH Rostock | https://www.labormedicus.de/ |
| Labor MVZ Westmecklenburg GbR | https://www.labor-schwerin.de/ |
| Landesamt für Gesundheit und Soziales, Rostock | https://www.lagus.mv-regierung.de |
| Landesamt für Landwirtschaft, Lebensmittelsicherheit und Fischerei Mecklenburg-Vorpommern | https://www.lallf.de |
| MVZ Labor Limbach Vorpommern-Rügen GmbH | https://www.labor-stralsund.de/ |
| MVZ Labor PD Dr. Volkmann und Kollegen Karlsruhe GbR | https://www.laborvolkmann.de |
| MVZ Martinsried GmbH | https://www.medizinische-genetik.de |

**Nucleic sampling, isolation and qRT PCR**

For the extraction of RNA from nasopharyngeal swabs following viral RNA isolation kits were used: NucleoMag VET (Macherey-Nagel, Germany), QIAamp Viral RNA (QIAGEN, Germany), Innuscreen DeltaPrep RNA-Virus Plus KFFLX (Analytic Jena, Germany), STARMag96 X4 Viral DNA/RNA 200 C (Seegene, South Korea) and/or TanBead (Tanbead, Taiwan). The following RT-PCR assays for the SARS-CoV-2 detection were applied: RealStar® SARS-CoV-2 RT-PCR Kit (Altona, Germany), RIDAGENE SARS-CoV-2 Lineage Kit (r-biopharm, Germany), GSD NovaType II SARS-CoV-2 Kit (Goldendiagnostics, Germany), Allplex SARS-CoV-2 Variant I and II Assay and Novaplex SARS-CoV-2 Variants I, IV and VII Assays  (Seegene, South Korea), cobas SARS-CoV-2 (Roche, Switzerland), BDMax SARS-CoV-2 and BD MAX SARS-CoV-2/Flu (Becton&Dickinson, USA), CerTest CoV2 + TNA-3 / 25 (CerTest, Spain) and/or Xpert Xpress SARS-CoV-2/SARS-CoV-2/Flu/RSV (Cepheid, USA).

**SARS-CoV-2 WGS using Nanopore sequencing**

cDNA was synthesized from isolated total RNA via a multiplexed amplicon-based nanopore sequencing protocol as previously described (https://www.protocols.io/view/ncov-2019-sequencing-protocol-v3-locost-bh42j8ye) and quantified by Qubit DNA HS kit (Invitrogen, USA). For sequencing of Delta and Omicron variants and their subvariants an adapted primer set using longer PCR products (V1200 Midnight) was used for full coverage of variant genomes. 50 ng of the amplicon pool was end-repaired, barcoded and ligated to the nanopore sequencing adapter following same protocol. Barcoded pools of 12 or 24 samples were loaded on a R9.4.1 nanopore flow cell (Oxford Nanopore Technologies – ONT, Oxford, UK) on a MinION Mk1B or Mk1C respectively.

**SARS-CoV-2 WGS using Illumina sequencing**

Libaries were synthesized either using the EEasySeq™ SARS-CoV-2 WGS Library Prep protocol (Nimagen, Netherland) or the Illumina COVIDSeq Test protocol (Illumina, USA) with Artic Primer v2. After library preparation, the barcoded pools of 96 samples were loaded on flowcells and sequenced on Illumina Nextseq 550 (High-Output Kit v2.5), Nextseq 2000 (with P2 or P3 reagents) or an Illumina NovaSeq 6000 instrument.

**Sequence analysis of Nanopore sequencing runs**

Data obtained from the MinION was analyzed using the ARTIC bioinformatics protocol (http://artic.network/ncov-2019). Briefly, the amplicon reads were basecalled using the ONT Guppy basecaller (v6.0.6) using the high-accuracy DNA model (dna_r9.4.1_450bps_hac) and afterwards demultiplexed with the ONT Guppy barcoder which is part of the Guppy basecalling suite (v6.0.6). Obtained reads per sample were filtered by read length between 300 and 1,100 nt for Artic V3 primer set and 250 and 2500 for the Artic Midnight Primer set. *Minimap2* was used for the alignment against the reference (MN908947.3). Subsequently, primer sequences were trimmed out and *Medaka* (r941_min_high_g360) was used for polishing and variant calling. Finally, *bcftools* was used for the consensus sequence generation.

**Sequence analysis of Illumina sequencing runs**

Sequence data obtained from the Illumina Nextseq 550 or Nextseq 2000 was analyzed using the minipipe, also known as the Cov pipeline (https://gitlab.com/RKIBioinformaticsPipelines/ncov_minipipe). Data generated on a NovaSeq6000 was analyzed using the DRAGEN COVID Lineage App from Illumina and Nextclade and Pangolin for the variant determination.

**Phylogenetic analyses** **and visualization**

For the phylogenetic and spatio-temporal analysis we included only sequences that fulfilled all filtering criteria defined above (N content of ≤ 10 %) and additionally were positively classified in clades by Nextclade and lineages by Pangolin. To the retained 3,493 sequences we added the Wuhan reference sequence and aligned them with MAFFT v7.490 [1] with default parameter. The resulting MSA was used to generate a corresponding phylogenetic tree using RAxML v.8.2.12 [2] with following parameters: model GTRGAMMA, 500 bootstraps, rapid bootstrapping, random number seed 32,323 for the ML search, random number seed 37,327 for the parsimony inference for the starting tree and as outgroup the reference sequence. This ML-tree was used as input for the spatio-temporal analysis. For the temporal analysis we subsequently added the sampling date information and generated a time tree with Augur refine tool with a coalescent skyline model and the reference as a root. For visualization purposes we exported the time tree with Augur export with JSON version v2.  For the visualization we used Auspice (Fig. 2A). Both Augur and Auspice are part of the Nextstrain project [3].

For the spatial analysis geographic coordinates were added via adaptive optical averaging of the patients’ zip codes. Data have been coarsened to gain sufficient anonymization. Geographical geojson vector maps of districts of Mecklenburg-Vorpommern were created with open data (http://opendatalab.de/projects/geojson-utilities/) provided by the Federal Agency for Cartography and Geodesy (https://gdz.bkg.bund.de/). Visualization was done using microreact (Fig. 3) [4].

For the spatio-temporal analysis of the regional 20J (Gamma) outbreak was used a subset of sequences that were classified as Gamma. With this subset median-joining networks were inferred using SplitsTree4 [5] with spring embedder iterations, excluding gapped sites and scaling nodes by taxa for an overview of the outbreak scenario and to identify potential outliers. The sampling date information was used as temporal trait and zip codes of patients as geographic coordinates. Phylogeographic continuous trait spatial Bayesian coalescent diffusion models were calculated for the subset of the 20J (Gamma) outbreak using BEAST (v.1.10.4) [6]. Convergence checked via Tracer (v.1.7.1) and time-scaled summary maximum clade credibility trees (MCC) with 10% post burn-in created using TreeAnnotator (v.1.10.4). The spatio-temporal diffusion models were analyzed and visualized using Spread (v.1.0.7) [7] and QGIS (v.3.16, QGIS.org) (Fig. S2).

1. Katoh K, Standley DM. MAFFT multiple sequence alignment software version 7: improvements in performance and usability. Mol Biol Evol. 2013;30(4):772-80. doi: 10.1093/molbev/mst010. PubMed PMID: 23329690; PubMed Central PMCID: PMC3603318.

2. Stamatakis A. RAxML version 8: a tool for phylogenetic analysis and post-analysis of large phylogenies. Bioinformatics. 2014;30(9):1312-3. doi: 10.1093/bioinformatics/btu033. PubMed PMID: 24451623; PubMed Central PMCID: PMC3998144.

3. Hadfield J, Megill C, Bell SM, Huddleston J, Potter B, Callender C, et al. Nextstrain: real-time tracking of pathogen evolution. Bioinformatics. 2018;34(23):4121-3. doi: 10.1093/bioinformatics/bty407. PubMed PMID: 29790939; PubMed Central PMCID: PMC6247931.

4. Argimon S, Abudahab K, Goater RJE, Fedosejev A, Bhai J, Glasner C, et al. Microreact: visualizing and sharing data for genomic epidemiology and phylogeography. Microb Genom. 2016;2(11):e000093. doi: 10.1099/mgen.0.000093. PubMed PMID: 28348833; PubMed Central PMCID: PMC5320705.

5. Huson DH, Bryant D. Application of phylogenetic networks in evolutionary studies. Mol Biol Evol. 2006;23(2):254-67. doi: 10.1093/molbev/msj030. PubMed PMID: 16221896.

6. Suchard MA, Lemey P, Baele G, Ayres DL, Drummond AJ, Rambaut A. Bayesian phylogenetic and phylodynamic data integration using BEAST 1.10. Virus Evol. 2018;4(1):vey016. doi: 10.1093/ve/vey016. PubMed PMID: 29942656; PubMed Central PMCID: PMC6007674.

7. Bielejec F, Rambaut A, Suchard MA, Lemey P. SPREAD: spatial phylogenetic reconstruction of evolutionary dynamics. Bioinformatics. 2011;27(20):2910-2. doi: 10.1093/bioinformatics/btr481. PubMed PMID: 21911333; PubMed Central PMCID: PMC3187652.
